# New Advanced Materials and Sorbent-Based Microextraction Techniques as Strategies in Sample Preparation to Improve the Determination of Natural Toxins in Food Samples

**DOI:** 10.3390/molecules25030702

**Published:** 2020-02-06

**Authors:** Natalia Casado, Judith Gañán, Sonia Morante-Zarcero, Isabel Sierra

**Affiliations:** Department of Chemical and Environmental Technology, E.S.C.E.T, Rey Juan Carlos University, C/Tulipán s/n, 28933 Móstoles, Madrid, Spain; natalia.casado@urjc.es (N.C.); judith.ganan@urjc.es (J.G.); sonia.morante@urjc.es (S.M.-Z.)

**Keywords:** natural toxins, food analysis, sample preparation, sorbent materials, microextraction

## Abstract

Natural toxins are chemical substances that are not toxic to the organisms that produce them, but which can be a potential risk to human health when ingested through food. Thus, it is of high interest to develop advanced analytical methodologies to control the occurrence of these compounds in food products. However, the analysis of food samples is a challenging task because of the high complexity of these matrices, which hinders the extraction and detection of the analytes. Therefore, sample preparation is a crucial step in food analysis to achieve adequate isolation and/or preconcentration of analytes and provide suitable clean-up of matrix interferences prior to instrumental analysis. Current trends in sample preparation involve moving towards “greener” approaches by scaling down analytical operations, miniaturizing the instruments and integrating new advanced materials as sorbents. The combination of these new materials with sorbent-based microextraction technologies enables the development of high-throughput sample preparation methods, which improve conventional extraction and clean-up procedures. This review gives an overview of the most relevant analytical strategies employed for sorbent-based microextraction of natural toxins of exogenous origin from food, as well as the improvements achieved in food sample preparation by the integration of new advanced materials as sorbents in these microextraction techniques, giving some relevant examples from the last ten years. Challenges and expected future trends are also discussed.

## 1. Introduction

Natural toxins are chemical substances naturally produced by living organisms (animal, plants or microorganisms) that are not toxic to them, but which can be potential health hazards to humans when ingested through food. These substances may naturally occur in food endogenously (toxic compounds that are implicit constituents of food resulting from the metabolism of a genus, species or strain, e.g., glycoalkaloids in potato or tetradotoxin in pufferfish) or exogenously (toxic compounds resulting from the metabolism of living organisms that occur in food as contaminants as they are not intentionally added, e.g., mycotoxins produced by molds grown in different products and toxins produced by algae that may be accumulated in edible marine organisms) [1,2]. The World Health Organization (WHO) encourages national authorities to monitor the most relevant natural toxins in the food supply. In this context, natural toxins of exogenous origin have received the most attention because of their potential harmful health risks and their involvement as natural contaminants. With respect to international organisms, these natural toxins of exogenous origin can be grouped in mycotoxins, phycotoxins (or marine toxins) and plant alkaloids [1,3,4]. Mycotoxins are toxic metabolites produced by certain types of molds, which can grow on a large number of foodstuffs such as cereals, dried fruits, nuts and spices. Most of these mycotoxins are chemically stable and survive food processing. The most common are aflatoxins (B1, B2, G1, G2 and M1), ochratoxins (A, B and C), patulin and fusarium toxins (deoxynivalenol, nivalenol, T-2 toxin, HT-2 toxin, zearalenone and fumonisins) [5]. On the other hand, marine toxins are produced during blooms of particular naturally occurring microalgae species in the ocean and fresh water. Thus, these toxins can be retained and bioaccumulated in shellfish and fish or contaminate drinking water. Their intake can be a potential hazard to consumers, since they are not eliminated by cooking or freezing, and might cause several adverse effects [6]. Conversely, in recent years, awareness about alkaloids of plant origin, such as pyrrolizidine, tropane and opioid alkaloids, has raised because of their occurrence as contaminants in different food products and the lack of data and knowledge about their exposure through food. These alkaloids are secondary metabolites of some plants, which can grow in fields as weeds and contaminate food crops appearing throughout the production of plant-derived products and finally be ingested, being toxic to humans [4,7,8,9,10,11,12]. The control of all these exogenous natural toxins in food is of high importance since they can cause from mild disorders (headache, vomiting, diarrhea, etc.) to serious situations (neurological disorders, carcinogenic, teratogenic or/and mutagenic effects, hepatic and renal damage, etc.) and can even be lethal. Moreover, they may cause the appearance of chronic diseases due to their harmful effects after a long-term exposure at high levels [1,2,3,4,5,6,7,8,9,10,11,12]. Therefore, food safety plays an essential role in reducing the risks related to the presence of harmful substances in food in order to protect consumers. In fact, the WHO in collaboration with the European Food Safety Authority (EFSA), the Food and Agriculture Organization (FAO) and the Codex Alimentarius Commission have established a legislation for mycotoxins and marine toxins [13,14], whereas pyrrolizidine, tropane and opioid alkaloids are in the process of being legislated, and at the moment only recommendations have been established for them [15,16,17]. In this sense, maximum residue limits (MRLs) for many of these natural toxins have been established in these guidelines to control the occurrence of these compounds in food [13,18].

Nonetheless, to achieve these limits and ensure the health of consumers it is important to develop high-throughput, sensitive and selective analytical methods to determine in a feasible way the presence of these natural toxins in foodstuffs [19]. However, the analysis of these compounds in food samples constitutes a challenging task because of the extreme complexity of these matrices, which considerably hinders the selective extraction of the target analytes and decreases the sensitivity of the method [20]. Despite significant advances in analytical instrumentation, particularly with respect to the combination of mass spectrometry and chromatographic separation, these techniques are not sensitive enough for direct analysis of complex matrices. Therefore, sample preparation is still a crucial step in food analysis in order to achieve an effective isolation and/or preconcentration of the analytes and provide an adequate clean-up of matrix interferences prior to instrumental analysis [21].

For many years, liquid-liquid extraction (LLE) and solid-phase extraction (SPE) have been the most extensively used sample preparation techniques. Due to the inherent drawbacks of LLE (such as: time-consumption, limited ability to extract polar compounds, requirement of large volumes of solvents, etc.), SPE has become more popular, as it provides more efficient recoveries and lower solvent consumption than LLE [22]. Nevertheless, current trends in sample preparation involve moving towards “greener” approaches by scaling down analytical operations and miniaturizing the instruments [23,24]. This has led in recent years to the development of different microextraction techniques for sample preparation procedures. In this sense, the SPE technique has been the axis of improving and creating even better and greener sorbent-based sample preparation techniques, which require less time and labor than SPE, such as: miniaturized solid-phase extraction (m-SPE), micro-dispersive solid-phase extraction (µ-dSPE), microextraction by packed sorbents (MEPS), pipette-tip solid-phase extraction (PT-SPE), solid-phase microextraction (SPME), stir-bar sorptive extraction (SBSE), and micro-solid-phase extraction (µ-SPE). These sorbent-based microextraction techniques have been proposed in recent years as an alternative to conventional sample preparation techniques to meet the Green Analytical Chemistry (GAC) requirements, as they involve advantages such as minimal solvent and sample consumption, fewer treatment steps, and reduction of waste generation [25]. Thus, they enable the development of cheaper, more cost-effective, and more environmentally friendly extraction and purification procedures.

On the other hand, the synthesis of new advanced materials for their application as sorbents in sample preparation has achieved considerable progress in the last decade, since these materials can play an important role in preconcentration processes and, in some cases, provide selective extraction of the target compounds [20,21,23,26,27]. Magnetic nanoparticles (MNPs), silica-based nanomaterials, metal-organic frameworks (MOFs), multiwalled carbon nanotubes (MWCNTs) and graphene oxide (GO) are currently the most used materials for the extraction of natural toxins from food samples, as they present large surface area and advanced physicochemical properties that enhance the efficiency, selectivity and sensitivity of the analytical procedures [21,27,28,29]. Additionally, the combination of these new materials with microextraction technologies enables the development of high-throughput sample preparation methods, which provide the advantages of both strategies leading to meet the GAC requirements and improving conventional extraction and clean-up technologies [23,30].

Some works in the literature have previously reviewed the determination of several natural toxins, such as phytotoxins [27] or mycotoxins [31], in food samples and other matrices. However, these works have just focused on one type of compounds but have not considered other natural toxins. On the other hand, other published reviews have addressed the development of new materials for their application to extract or detect chemical contaminants in order to ensure food safety [27,32,33]. Nevertheless, these revisions have not specifically evaluated the integration of these new materials in microextraction procedures for the particular determination of natural toxins in food. Accordingly, this review aims to provide an overview of the different sorbent-based approaches employed for the microextraction of natural toxins in food products, as well as the improvements achieved in sample preparation by the integration of new materials as sorbents in these microextraction techniques, by giving some relevant examples from the last ten years (from 2009 to 2019). The criteria of microextraction followed in this review are the use of the sorbent-based microextraction technique and the reduced amount of sorbent employed to achieve the extraction of the target analytes. Nevertheless, other aspects, such as the amount of sample and the volume of solvents, are also indicated. Natural toxins of exogenous origin that have been legislated or in the process of regulation, like mycotoxins, marine toxins, pyrrolizidine alkaloids, tropane alkaloids and opioid alkaloids, have been established as featured analytes due to the harmful risk their intake may entail on human health, as well as their possible regular consumption through diet. Challenges and expected future trends are also included.

## 2. Sorbent-Based Microextraction of Natural Toxins from Food Samples

The miniaturization of conventional sample preparation procedures has been proposed as an alternative for developing analytical methods with improved analytical characteristics (accuracy, precision, sensitivity, etc.) along with a decrease in sample and solvent consumption, reduction of hazardous reagents and wastes, and saving energy and time. As a result, new formats and configurations have arisen to carry out microextraction procedures, which overcome drawbacks of conventional techniques. Table 1 collects the most relevant works published in the last decade dealing with microextraction techniques based on sorbent-adsorption, which have been applied for the isolation of natural toxins from different food samples. In this sense, Solid-Phase Microextraction (SPME) has been the most popular [34,35,36,37,38]. However, procedures based on the dispersion of the sorbent material, such as micro-dispersive solid-phase extraction (µ-dSPE) and micro-solid-phase extraction (µ-SPE) have also been used [39,40,41]. All the works reviewed were performed for the analysis of mycotoxins (ochratoxins, aflatoxins, zearalenone, fumonisins and patulin) in different food matrices (mainly, wine, cereals and nuts). Only three of the methodologies developed in these articles perform the simultaneous determination of different types of mycotoxins [37,39,40], while the other works only described the individual determination of a specific analyte [34,35,36,38,41,42]. Concerning detection mode, mass spectrometry (MS) and fluorescence detection (FLD) were the techniques employed to detect these natural toxins (Table 1). Most of these works used MS detection, which is the most suitable technique to detect the presence of contaminants in food at trace levels thanks to its high sensitivity and to its structural elucidation capability, which enables the unequivocal identification and confirmation of the target analytes. In contrast, the FLD also provides high sensitivity and selectivity, but if the analytes do not show fluorescence it is necessary to carry out a derivatization process (pre-column or post-column derivatization) for their detection, which can sometimes be time consuming.

### 2.1. Solid-Phase Microextraction (SPME)

Solid-Phase Microextraction (SPME) was first developed by Pawlisyn and Arthur in 1990 [43] to overcome some inherent limitations of SPE, such as saving time and reducing the consumption of organic solvents. This technique is a solvent free sample preparation strategy which consists in a fused silica fiber coated with a suitable stationary phase attached to a modified micro-syringe. Its configuration enables easy access of the analytes, enhancing their extraction and desorption. SPME presents many advantages, it is simple, sensitive, solvent-less, time-efficient (combines sampling and extraction in one step) and can be easily coupled with analytical separation instruments such as HPLC, gas chromatography (GC) and capillary electrophoresis (CE). Additionally, it can be applied to liquid, gas and solid samples. However, it also presents some drawbacks: batch to batch variation, fragility of fibers, restricted choice of commercially available stationary phases and fiber coatings, and insufficient selectivity of these materials [25]. Nevertheless, despite these negative aspects, due to its versatility and benefits it is easy to find a large number of papers which use SPME to perform sample preparation and analyte enrichment. Regarding the application of SPME in recent years in the extraction of natural toxins from food samples, it has been used to extract ochratoxins, aflatoxins, and patulin from different foods, such as drinks, cereals, nuts, dried fruit and spices (Table 1). For instance, Quinto et al. proposed a SPME procedure to extract four aflatoxins from cereal flours followed by its subsequent determination by HPLC-FLD with post-column photochemical derivatization step to improve the limits of detection (LODs) and enhance the native fluorescence of aflatoxins B1 and G1 [34]. Different commercial fibers were evaluated, with polydimethylsiloxane/divinylbenzene (PDMS/DVB) being the one with the greatest extraction capability. Also, other parameters, such as temperature, pH, ionic strength, adsorption and desorption time and mobile phase, were studied and optimized. Nevertheless, under the optimized conditions low recovery values were achieved, ranging from 49% to 59%, and LODs were between 0.035–0.2 µg/Kg.

**Figure 1:**
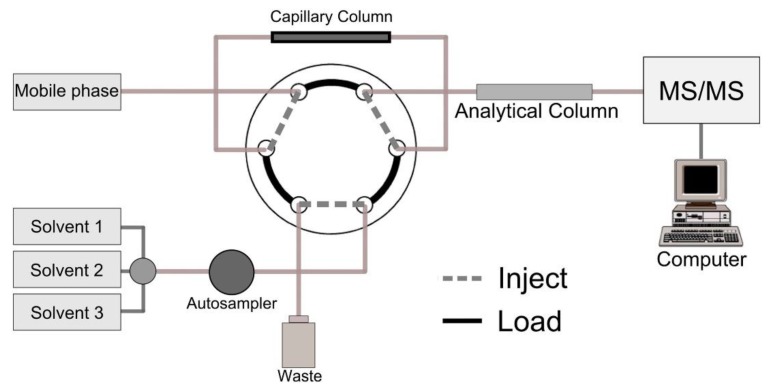
General view of the schematic setup utilized for packed in-tube solid-phase microextraction (SPME) system (reprinted with permission from reference [38]).

A variant of this method is the in-tube SPME technique, which involves employing internally coated open-tubular capillary columns or needles, instead of using coated fibers. This SPME modality arose with the aim of easily coupling SPME on-line with HPLC or GC [44]. In addition, in-tube SPME can overcome some drawbacks related to conventional SPME, such as: fragility, low sorption capacity and extraction phase bleeding. Additionally, it reduces analysis time and provides better accuracy, precision and sensitivity. Nonetheless, it requires more complex instrumentation than conventional SPME. In this context, Kataoka et al. developed different in-tube SPME methods for the extraction of several mycotoxins (aflatoxins, patulin and ochratoxins) from different food matrices (nut, cereals, dried fruits, spices, fruit juice) prior to their analysis by HPLC-MS [35,36,37]. In these works, a capillary GC column was used as the in-tube SPME device, which was placed between the injection loop and the injection needle of the autosampler (Figure 1). The procedures were carried out in the draw/eject extraction mode, which means the autosampler has to be programmed to control the extraction, desorption and injection steps of SPME. For this purpose, a programmable autosampler with a metering pump is required, which limits its applicability, since it requires specific instrumentation. The extraction of analytes onto the capillary coating was performed by repeated draw/eject cycles of the sample in the load position of the autosampler. The repeated draw/eject cycles were carried out in the same vial including the sample. Afterwards, desorption of the extracted analytes from the capillary coating to the analytical column was performed by switching the injector to the inject position. In all these works, different types of coated capillary columns were tested. Best extraction performance was achieved using porous divinylbenzen polymer (Supel-Q PLOT) and carboxen molecular sieves polymer (Carboxen-1006 PLOT) capillary columns for aflatoxins [35] and both patulin and ochratoxins, respectively [36,37]. Under the optimized conditions, recovery values for aflatoxins were in the range 81–109%, and their chromatographic separation was achieved within 8 min with LODs values of 0.0021–0.0028 µg/L [35]. Therefore, this method is more suitable and effective than the previous work reported by Quinto et al. for the analysis of aflatoxins [34], since higher recovery values and sensitivity are achieved with similar run-time analysis in the chromatographic separation [35]. On the other hand, high recovery values were also achieved with in-tube SPME for patulin (>92%) [36] and ochratoxins A and B (>88%) [37], with LODs of 0.023, 0.092 and 0.089 µg/L, respectively. In addition, the chromatographic separation of these compounds was achieved within 5 min [36,37].

More recently, Andrade et al. proposed an in-tube SPME method using a capillary packed with C18 particles in the flow-through extraction mode instead of the draw/eject extraction approach for the extraction of ochratoxin A from wine samples and its subsequent analysis by HPLC-MS/MS [38]. The flow-through extraction mode is a more versatile approach since it can operate in manual and automated sample introduction, whereas the draw/eject approach can only be performed in automated mode. Moreover, the flow-through extraction mode is more suitable for packed and monolithic capillary columns, since it overcomes problems of high backpressure, which may occur when using these types of capillaries in the draw/eject extraction mode. In addition, the flow-through extraction mode exhibits better extraction efficiency in a shorter time than the draw/eject approach [38,44]. Nevertheless, the in-tube SPME extraction proposed by Andrade et al. [38] with the flow-through extraction mode provided lower recovery values for ochratoxin A (61–73%) than the ones previously reported by Kataoka et al. using the draw/eject extraction mode (>88%) [37]. On the other hand, the LOD report for ochratoxin A was similar (0.02 µg/L) [38], but the chromatographic separation took longer (11 min) than the method proposed by Kataoka et al. (5 min) [37].

### 2.2. Micro-Dispersive Solid-Phase Extraction (µ-dSPE)

µ-dSPE is a simple miniaturization of the conventional dispersive solid-phase extraction (dSPE) procedure in which small amounts of sorbents (lower than 100 mg) and solvents are used to extract and enrich compounds from different matrices. In contrast to SPE, in this method, the sorbent is dispersed into the sample solution, providing a more effective interaction between the analyte and the sorbent that improves extraction, since it avoids passing the sample extract through a packaged cartridge. After the elution step, the sorbent can be separated by centrifugation or filtration. The easy operation of µ-dSPE makes this technique very suitable for performing multicomponent extraction of different analytes saving time and reagents. For instance, it has been used to simultaneously extract six estrogenic mycotoxins (zearalenone, α-zearalenol, β-zearalenol, zearalanone, α-zearalanol and β-zearalanol) from mineral water and powdered infant milk using multiwalled carbon nanotubes (MWCNTs) as sorbent prior their determination by HPLC-MS [39]. Similarly, Du et al. developed a method combining microwave-assited µ-dSPE and ultra-high performance liquid chromatography coupled to tandem MS (UHPLC-MS/MS) to determine 6 different mycotoxins (fumonisin B1, aflatoxin B1, ochratoxin B, T-2 toxin, ochratoxin A and zearalenone) in different food matrices, including peach seed, milk powder, corn flour and beer, using zirconia nanoparticles as sorbent [40]. In both works, several experimental parameters affecting the extraction efficiency (pH, type and amount of sorbent and elution solvent, etc.) were evaluated and optimized, and both methods were successfully validated, achieving good recovery values. With 80 mg of MWCNTs, recoveries ranged from 77% to 120%, and limits of detection (LODs) were in the range 0.05–2.02 µg/L [39]. However, zirconia nanoparticles proved to be very superior sorbents, since with a really small amount of them (12.5 µg) and a very short extraction time (2 min) the recoveries of the different target mycotoxins ranged between 84 and 105% with lower LODs (0.0022–0.017 µg/L and 0.0036–0.033 µg/Kg for liquid and solid samples, respectively) [40] than the ones described for the previous work [39]. Using this minimum amount of sorbent was possible because the authors previously dispersed 2 mg of the zirconia nanoparticles in an aqueous solution, and then an aliquot of the dispersed solution containing 12.5 µg of the nanoparticles was used to perform microextraction of the target analytes, showing the great extraction potential of this type of sorbent.

### 2.3. Micro-Solid-Phase Extraction (µ-SPE)

**Figure 2:**
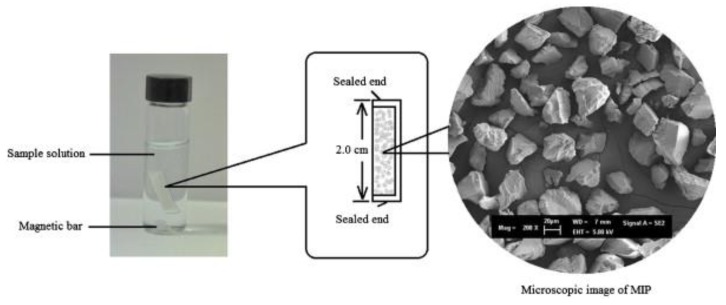
Schematic diagram and microscopic image of molecularly imprinted polymer (MIP) sorbent used in micro-solid-phase extraction (µ-SPE) for the extraction of ochratoxin A (reprinted with permission from reference [41]).

The sorbent-based µ-SPE technique emerged as an alternative to the multi-step SPE procedure, as the clean-up and extraction step are performed simultaneously. In this procedure the sorbent is enclosed in a porous membrane sheet, the ends of which are heat-sealed. This membrane is directly placed into the sample solution under stirring, and due to its porosity, analytes diffuse freely and are extracted by the sorbent. Afterwards, the membrane is removed from the solution and immersed into the elution solvent to perform the desorption of analytes assisted by ultrasonification. Lee et al. developed a µ-SPE strategy to extract ochratoxin A from coffee and grape juice [41]. To achieve higher selectivity, they used a commercial molecularly imprinted polymer (MIP) as sorbent material which was packed in the membrane sheet device (Figure 2). After extraction, the sample extract was analyzed by HPLC-FLD within 4 min. As ochratoxin A presents natural fluorescence, a derivatization step was not necessary. Under the optimized conditions, recoveries ranged from 91% to 101% and low limits of detection (LODs) were achieved (0.02–0.06 µg/Kg). Despite using FLD, these LOD values are similar to the ones achieved by MS [40], probably because using a MIP as sorbent provides higher clean-up of matrix interferences. Thus, it may increase not only the selectivity, but also the sensitivity of the analytical method. The proposed method [41] was applied to the determination of ochratoxin A in real samples. Totals of 62% and 91% of the coffee and grape juice samples assayed, respectively, were contaminated with this mycotoxin. Nonetheless, the levels found of ochratoxin A were below its legal maximum residue limits (MRL).

### 2.4. Microextraction by Packed Sorbent (MEPS)

**Figure 3:**
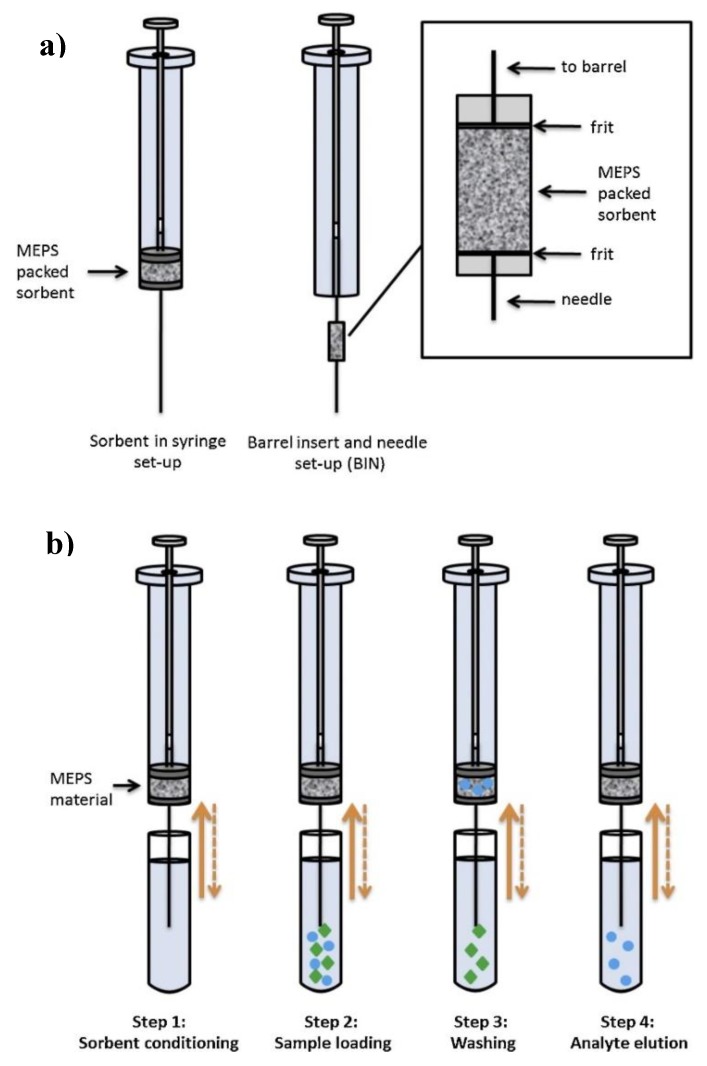
Microextraction by packed sorbent (MEPS) in syringe set-up and barrel insert and needle (BIN) set-up (**a**), typical MEPS procedure steps (**b**) (reprinted with permission from reference [22]).

The Microextraction by Packed Sorbent (MEPS) procedure was created in 2004 as a miniaturization of conventional SPE by Abdel-Rehim et al. in order to reduce sample and solvent volumes and provide an automated method through its easy interface to chromatographic systems [45]. In this technique, the sorbent (1–4 mg) is integrated into a micro-syringe needle and not in a separate column, as in SPE. Additionally, unlike SPE, in MEPS there is a two-directional flow potential (up and down), which provides repetition of each step, enhancing the sample-sorbent contact and improving the method’s efficiency (Figure 3). Nevertheless, the main drawback of this technique is its limitation in terms of being applied to viscous or highly concentrated samples, which need to be previously diluted in order to avoid flow blocking through the device. For this reason, this technique is more suitable for sample preparation of liquid matrices. For instance, MEPS has been used for the extraction of ochratoxin A from wine samples using a C18 sorbent prior to its determination by HPLC-FLD [42]. Different experimental parameters, such as the sample and elution solvent volumes, the number of extraction cycles and the flow speed were optimized. Wine samples were previously diluted with an acidified aqueous solution, probably to avoid the negative influence of ethanol on the extraction efficiency which may directly eluted the analytes during the loading step, as reported by other authors [46,47]. Under optimal conditions, an aliquot of 50 μL of diluted sample was passed through the C18 sorbent 7 times, and elution was achieved with 50 μL of Acetonitrile (ACN)/2% aqueous acetic acid solution (90/10, *v*/*v*), which were directly analyzed in the chromatographic system in less than 6 min. Recovery values ranged between 76–108% and the LOD was 0.08 µg/L. Additionally, the proposed MEPS extraction was compared with well-established SPE and immunoaffinity clean-up (IAC) methods. The precision of the MEPS procedure was similar to the IAC one, whereas SPE was clearly the one with the worst reproducibility. The applicability of the proposed MEPS strategy was shown by te analysis of 60 different wine samples, requiring 15 min of extraction and 15 min of chromatographic analysis for each sample. The possibility of automating the procedure proved the suitability of MEPS when a significant number of samples have to be analyzed in short time, such as in regular monitorization of food quality and safety.

## 3. Integration of New Advanced Materials as Sorbents on Microextraction Techniques to Isolate Natural Toxins from Food Samples

Sometimes, the commercially available microextraction techniques and sorbent materials used limit the development of the analytical methodologies. One of the crucial parameters that determine success of sample preparation is the choice of the sorbent material. Depending on the analytes to be extracted, the sorbent material must have specific characteristics that allow obtaining the highest extraction efficiency. In addition, using minimal amounts of sorbents is one of the requirements of the Green Analytical Chemistry (GAC) when developing an analytical procedure [29]. Thus, the sorbent must have advanced functional properties to be able to potentially interact with the target analytes to achieve high extraction efficiency by using minimal amounts of it. In this sense, current trends in the development of analytical methods are focused on the synthesis of new advanced materials to apply them as sorbents in sample preparation procedures. Among these materials, magnetic nanoparticles (MNPs), silica-based nanomaterials, metal-organic frameworks (MOFs), multiwalled carbon nanotubes (MWCNTs) and graphene oxide (GO) have been the most employed for the extraction of natural toxins from food products (Table 2). The advanced properties of these materials, such as their large surface area, low resistance to diffusion, fast sorption kinetics and large adsorptive capability make them very suitable for sample preparation, as they improve the efficiency, selectivity and sensitivity of the analytical procedures. Moreover, the integration of these new materials in microextraction technologies enables developing high-throughput analytical methods with the advantages of both strategies. Thanks to this integration, conventional and commercially available procedures can be improved and GAC requirements can be accomplished. In this sense, in the last decade, different new materials have been used to extract natural toxins from food products by their combination with different microextraction techniques, such as m-SPE, in-syringe SPE, PT-SPE, µ-dSPE, µ-MSPE, µ-SPE, SPME and SBSE (Table 2). They have proved their efficiency in the extraction of several mycotoxins (mainly aflatoxins, ochratoxins, patulin and zearalenone) and marine toxins, which have been mainly extracted from cereals, drinks, dairy products and seafood (Table 2). Sometimes, these sorbent materials lack or have little selectivity during the extraction procedure, leading to the extraction of matrix interferences along with the analyte that may hinder its detection. To overcome this problem, MIPs can be synthesized as sorbents by polymerization processes [30]. In this sense, different MIPs have been applied in m-SPE, µ-MSPE and SBSE for the extraction of patulin, T-2 toxin, fumonisin, aflatoxins and ochratoxins from food samples (Table 2). Nevertheless, when developing multicomponent methods to simultaneously extract different compounds belonging to different chemical families in a single run, the lack of selectivity of the materials is desirable, since in this case the sorbent must be able to extract a wide range of compounds. Therefore, in these cases specificity is not required. On the other hand, the analytical procedures published in the last decade, which integrate new materials in microextraction techniques for the extraction of natural toxins from food, have been mainly combined with the detection of analytes by HPLC coupled to MS or FLD, and to a lesser extent with ultraviolet detection (UV), such as the diode array detection (DAD) (Table 2). In contrast, there are no works using GC as a separation technique instead of HPLC. Indeed, for the analysis of these natural toxins, it is more suitable to use HPLC, since they are not very volatile compounds. Therefore, sometimes, to achieve their analysis by GC it is necessary to perform a derivatization process, which is more complex and time consuming than the determination by HPLC.

### 3.1. Miniaturized Solid-Phase Extraction (m-SPE)

Miniaturized SPE is almost equal to conventional SPE, with the same operation steps (conditioning, sample loading, washing and elution). The miniaturization concept just involves scaling down the amount of sample, sorbent and/or solvents used, but the sorbent still packed inside the extraction cartridge between two frits. Among researchers, there are different criteria as to what amount of sorbent or elution volume should be used to be considered a miniaturized SPE. Due to the great diversity of opinions, this review considers those works employing an amount of sorbent below 100 mg. Commercial packed cartridges for SPE are usually available with high amounts of sorbents (100–500 mg). To miniaturize the extraction, many authors have developed new materials, not only to pack their own cartridges, but also with the aim of designing potential sorbents for sample preparation with specific characteristics, which improve extraction efficiency using a minimal amount of them.

In this context, Liu et al. proposed the synthesis of supramolecular hyperbranched polymers as sorbents, which provide molecular capture through available non-covalent interactions (hydrogen bonding and π-π interactions) [48]. These polymers were tested as sorbents to extract four aflatoxins from cereal powder samples (rice, oatmeal and pear barley). Under the optimal experimental conditions, with 50 mg of one of these polymers the recovery values of the target aflatoxins were in the range of 83–103%. Moreover, under the same conditions, the sorbent was compared with a homemade C18 sorbent, which is a widespread commercially available SPE sorbent. The polymer prepared proved to have superior extraction efficiency than the traditional C18 sorbent. Although the extraction efficiency towards aflatoxins G1 and G2 was similar between both sorbents, the polymer was significantly superior in the extraction of aflatoxins B1 and B2, due to its major sites of hydrogen bonding and π-electronic stacking interactions. In contrast, 50 mg of C18 sorbent was insufficient to achieve successful extraction of aflatoxins B1 and B2. Thus, this highlights the limited extraction capacity of commercial sorbents when using small amounts of them.

On the other hand, Appell and Jackson proposed the preparation of cyclodextrin (CD)-based polymers as SPE sorbents to extract patulin from apple juice samples [49]. The incorporation of CDs into polymers results in a porous material with cavities, which can act as potential binding sites for analytes. It was observed that with 30 mg of polyurethane-β-CD polymer used as sorbent it was possible to successfully extract the target analyte from the food matrix, thanks to the hydrogen bonding interactions established between the hydroxyl groups of β-CD and patulin.

In contrast, other authors have developed more selective strategies to achieve the extraction of patulin through the preparation of MIP materials [50,51]. In this sense, Anene et al. developed a MIP sorbent by bulk polymerization using patulin as template [50]. The material was used in SPE for the extraction of patulin from apple and juice samples prior to its determination by HPLC-DAD analysis. Under the extraction conditions assayed, 50 mg of the MIP were established as the optimal sorbent amount, showing high selectivity and great affinity towards patulin providing recovery values in the range 82–98%. To show its applicability, the proposed method with the patulin MIP was used for the analysis of different food samples (juices and apples). Surprisingly, patulin was found in three samples exceeding its MRL fixed by the European Commission regulations [13]. This could be due to template bleeding, which is one of the inherent problems of MIP technology. During MIP’s preparation, sometimes it may be difficult to completely eliminate the template molecules. Thus, if some residues of the template remain in the sorbent, it can lead to false positive results. Nonetheless, this problem can be solved by using dummy templates, which involves replacing the template with another molecule with an analogous structure to the target analytes. Accordingly, Zhao et al. prepared a dummy MIP by precipitation polymerization for the extraction of patulin using 2-oxindole and 6-hydroxynicotinic acid as dual-dummy template molecules [51]. In this case, with a smaller amount of sorbent (30 mg) than the previous work [50], it was possible to successfully extract patulin obtaining high recovery values (81–106%). Moreover, the extraction efficiency of the method proposed with the dummy MIP was compared with the QuEChERS method, showing better purification effect and higher recovery of patulin. Also, the method proposed by Zhao et al. [51] was more sensitive (LOD were 0.05–0.2 µg/Kg) than the one previously described by Anene et al. (LOD 8.6 µg/L) [50]. This was mainly due to the fact that the former used HPLC-MS/MS, whereas the latter used HPLC-DAD, which is inherently a less sensitive detection technique. Moreover, the mobile phase composition of the chromatographic method was the same in both works (water and ACN). However, Zhao et al. [51] used a gradient elution with a linear ramp, which increased the composition of ACN from 5% to 90% in 2 min, while the chromatographic analysis of Anene et al. [50] was performed in isocratic mode (water: ACN (90:10, *v*/*v*)). The retention time of patulin was shorter using the gradient elution mode (4.27 versus 5.18 min). In addition, when the dummy MIP was applied for the analysis of different food matrices (apple, apple juice, hawthorn, hawthorn juice, mixed juice, wines and tomato) no patulin residues were detected in them [51]. Therefore, the use of dummy MIPs might be more reliable to avoid false positive results.

On the other hand, MIPs have also been developed by bulk polymerization for the selective extraction of other mycotoxins (fumonisins and T-2) from different food samples [52,53]. In both works, the selectivity of the MIPs synthesized was evaluated through a cross-reactivity study, which involved performing the extraction procedure of the target analytes in the presence of other compounds, which may also interact with the sorbent when they occur together in the sample with the analytes. Moreover, the extraction efficiency of the MIPs developed was compared with the results obtained when using other popular commercial sorbents, such as C18, strong anion exchange (SAX), IAC and Oasis HLB® sorbents. These commercial sorbents were packed with a higher amount (200–500 mg) of sorbents than the amount used to perform miniaturized SPE with the MIPs materials (20 and 50 mg for extraction of fumonisins [52] and T-2 [53], respectively). In the case of the MIP developed for the selective extraction of fumonisins [52], recoveries varied from 62% to 86%. In contrast, higher recoveries were achieved with SAX (71–111%), while low recoveries were obtained with C18, which in addition did not show selectivity towards fumonisins at all. Despite the SAX sorbent showed the highest extraction efficiency, it showed less selectivity towards fumonisins than the MIP, since SAX has selectivity towards fumonisins and any compound with a negatively charged carboxylic group. Regarding the MIP designed for the selective extraction of T-2 [53], the recovery values obtained varied from 60 to 73%. In contrast, recoveries were in the range of 74–104% and 60–85% when using Oasis HLB^®^ and IAC sorbents. Nevertheless, as in the previous work [52], although the highest extraction efficiency was achieved with Oasis HLB^®^, the MIP and the IAC materials were superior regarding selectivity and providing less matrix effects.

To carry out the simultaneous extraction of several compounds with different nature, the most suitable thing is to use non-specific materials with potential advanced characteristics, which can be able to efficiently extract a wide range of compounds. For instance, graphene, which is a carbon nanomaterial, has proved to be a potential sorbent material because of its advanced properties, such as its huge surface area (>2000 m^2^/g), good chemical and mechanical stability, good adsorption capability and possibility of functionalization, among other things. In this context, a reduced GO and gold nanoparticle nanocomposite was synthesized and applied as sorbent for simultaneous purification and pre-concentration of 9 different mycotoxins (including aflatoxin B1, aflatoxin M1, ochratoxin A, zearalenone, α-zearalenol, β-zearalenol, zearalenone, α-zearalanol, β-zearalanol) from milk samples prior their analysis by UHPLC-MS/MS [54]. Different experimental parameters of the procedure were optimized. Best extraction conditions were achieved by only using 10 mg of the sorbent material, using a mixture of methanol/ACN/formic acid (50/49/1, *v*/*v*/*v*) as elution solvent. Under these conditions, recoveries achieved for the target analytes ranged from 70% to 111% and low LOD values were obtained (0.01–0.07 µg/L). Moreover, the separation of the 9 mycotoxins was achieved in a relatively short time (within 9 min) thanks to the use of UHPLC, which enables to significantly reduce analysis time by using short chromatographic columns packed with sub-2 µm particles and high-pressure conditions (1000 bar).

### 3.2. In-Syringe Solid-Phase Extraction

**Figure 4:**
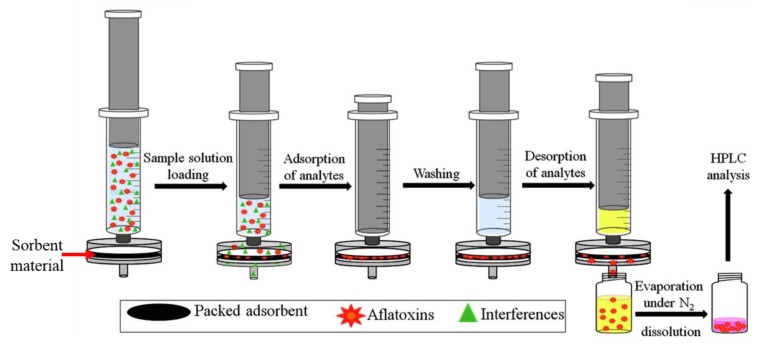
Schematic illustration of the in-syringe solid-phase extraction procedure using aflatoxins as target analytes (adapted and reprinted with permission from reference [57]).

In-syringe SPE is a novel miniaturized variant of the conventional SPE method, which involves placing a minimal amount of sorbent inside a syringe holder, which is connected to the syringe tip (Figure 4). The procedure steps are the same than in SPE. This setup, besides increasing the contact area between the sorbent and the analytes, enables the use of higher sample loading flows than conventional SPE cartridges, improving the extraction efficiency.

**Figure 5:**
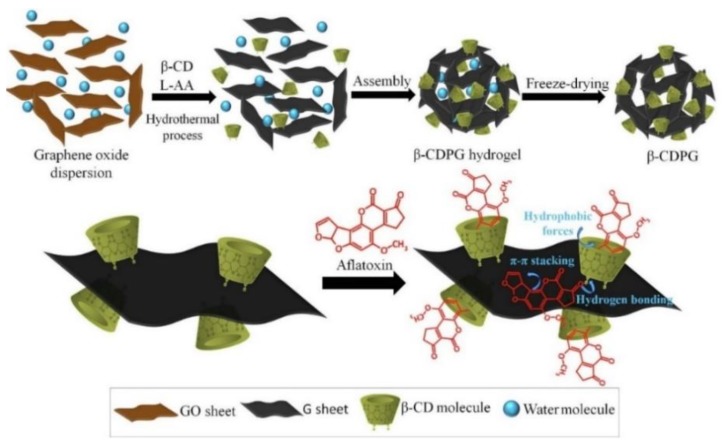
Schematic representation of the process to prepare porous graphene functionalized with β-CD and its interactions with a typical aflatoxin molecule (adapted and reprinted with permission from reference [57]).

In the determination of natural toxins in food samples, this technique has only been used for the extraction of aflatoxin from corn and soybean products [55,56,57]. Different types of materials based on graphene have been evaluated for this purpose: magnetic three-dimensional graphene nanoparticles fixed on a perforated magnetic sheet [55], electrospun polyurethane nanofibers doped with GO [56] and porous graphene functionalized with β-CD [57]. In all these works, successful results were achieved, since recovery values were in all cases higher than 75%, showing the great potential of these materials to extract the target analytes. All the in-syringe SPE procedures proposed for the extraction of aflatoxins were combined with the analysis by HPLC-FLD. LODs were the same for the methods using the magnetic three-dimensional graphene nanoparticles and electrospun polyurethane nanofibers doped with GO (0.09–0.15 µg/Kg) [55,56]. However, the nanoporous graphene material functionalized with β-CD (Figure 5) proved to be more sensitive, as lower LODs were achieved (0.0075–0.03 µg/Kg) [57]. Nonetheless, all three materials showed high extraction efficiency and great potential as sorbents. On the other hand, the magnetic material could have probably taken advantage of its magnetic nature using a simpler method like MSPE, by dispersing the material in the sample extract and separating it after with a magnet, instead of placing it in the syringe holder.

### 3.3. Pipette-Tip Solid-Phase Extraction (PT-SPE)

The Pipette-Tip Solid-Phase Extraction (PT-SPE) technique has also emerged as a miniaturized version of conventional SPE, in which the sorbent material is packed inside plastic micropipette tips. In this sense, extraction is carried out by repeated aspirating/dispensing cycles of the sample solution using pipettor with a single channel or multichannel. Shen et al. proposed a PT-SPE method using graphene as sorbent material for the extraction of seven lipophilic marine toxins, including yessotoxins, okadaic acid, dinophysitoxin-1, gymmodimine, spirolides-1, pectenotoxin-2 and azaspiracid-1 from shellfish tissues [58]. For the PT-SPE procedure, GO was first synthesized, and afterwards it was reduced by hydrazine to obtain graphene, which was packed (2 mg) into a 200 µL pipette tip. Extraction was performed by 20 repeated aspirating/dispensing cycles, discarding the eluate as waste, and then analytes were eluted by 10 repeated aspirating/dispensing cycles. The resultant eluate was then injected in the HPLC-MS/MS system. Separation of the seven compounds was achieved within 5 min and high recovery values were obtained (78–90%). The results were compared with the ones obtained with different commercially available sorbents (C18, HLB, Strata-X and MWCNTs) using the same extraction procedure. Nevertheless, graphene proved its great potential as a sorbent due to its ultrahigh surface area which provides high loading capacity. Recoveries obtained with Strata-X were very similar to the ones achieved with graphene, whereas the other sorbents were less effective, proving the superiority of graphene. The proposed PT-SPE method was successfully validated, obtaining LODs ranging from 0.1 to 1.5 µg/Kg, and it was applied for the analysis of 67 mussel samples in China. Okadaic acid was found to be a dominant contaminant and yessotoxins were detected at low level in the samples of one of the geographic areas evaluated.

### 3.4. Micro-Dispersive Solid-Phase Extraction (µ-dSPE)

GO, which is cheaper and exhibits similar adsorption capacity than graphene, has been used as sorbent in the µ-dSPE of aflatoxins from peanuts [59]. GO was first synthesized and characterized as sorbent. Afterwards, a small amount of the prepared material was dispersed into the sample solution to perform the extraction of the target analytes. Different parameters that could affect the extraction efficiency of the procedures (GO amount, elution solvent, extraction and desorption time, etc.) were evaluated and optimized. The results suggested that GO was a successful sorbent material. Under optimal conditions, 5 mg of GO were enough to achieve the extraction of four aflatoxins from peanut samples obtaining recovery values ranging from 85% to 101%. Aflatoxins were analyzed from the purified sample extracts by HPLC-FLD. For this purpose, before column separation a derivatization step was required. The four aflatoxins were separated within 25 min, which is a longer time than the one reported by other authors [34] and LODs ranged from 0.08 to 0.65 µg/Kg. 

### 3.5. Micro-Magnetic Solid-Phase Extraction (µ-MSPE)

Undoubtedly, in recent years, the preparation of new magnetic materials has been the most popular trend in sample preparation, as can be seen from the large volume of articles published on the subject (Table 2). In recent years, MSPE has attracted significant attention because of its simplicity and superiority over conventional methods. In addition, the high potential of the magnetic materials has enable reducing the amounts of sorbents and solvents in the extraction procedures, giving rise to the appearance of its miniaturized version µ-MSPE. This technique is very similar to µ-dSPE, since it is based on the dispersion of the magnetic sorbent into the sample solution. However, it differs in the way of separating the sorbent from the sample solution. Its magnetic properties allow the removal of the sorbent enriched with the target analytes by using an external magnet, instead of using centrifugation or filtration as in µ-dSPE. In this way, the procedure is simplified, reducing the use of additional steps, decreasing sample manipulation and saving time.

MNPs involve having a magnetic core, which in most cases is magnetite (Fe_3_O_4_), which is coated with different organic ligands to provide the material with specific functional characteristics. In this sense, many types of MNPs have been used as sorbents for the extraction of natural toxins from food samples. For instance, MNPs coated with polydopamine have been synthesized and successfully applied as sorbents for the extraction of zearalenone-related compounds from dairy products [60] and drinking water [61], as well as for the extraction of aflatoxins from red wine [62]. In this last work, a small amount of polydopamine-coated MNPs (4.4 mg) was used as sorbent [62].

**Figure 6:**
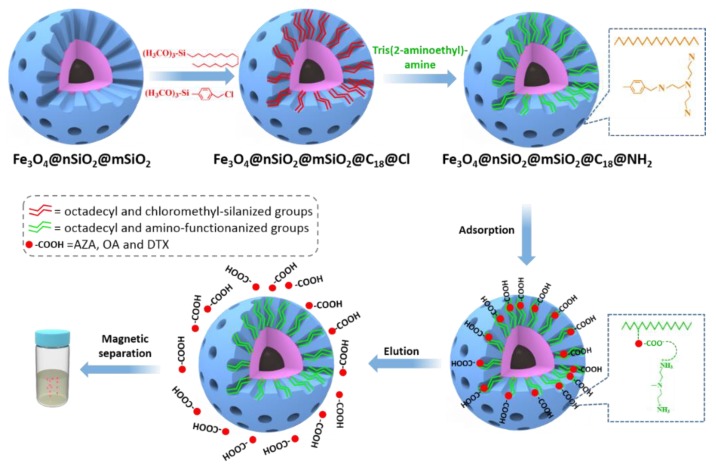
Preparation scheme of magnetic mesoporous silica nanoparticles bifunctionalized with octadecyl (C18) and amino (NH_2_) and enrichment process of azaspiracids (AZA), okadaic acid (OA) and dinophysistoxins (DTX) (Reprinted with permission from reference [64]).

On the other hand, another common coating material of MNPs is silica, which in turn, due to the presence of silanol groups in its surface, it can be functionalized with different organosilanes and/or affinity ligands which enable the selective extraction of analytes. In this context, aptamer-functionalized silica MNPs have been proposed as sorbents for the extraction of aflatoxin M1 from dairy products [63]. More recently, magnetic mesoporous silica nanoparticles have been bifunctionalized with octadecyl (C18) and amino (NH_2_) groups in order to obtain a magnetic mixed-mode sorbent with reversed-phase/weak anion exchange interactions [64]. This novel and advanced material (Figure 6) has been successfully applied for the extraction of six lipophilic marine toxins from shellfish samples, with recovery values ranging from 83% to 119% using 50 mg of sorbent. MWCNTs have also been used as coating of MNPs for the extraction of zearalenone-related compounds from maize samples [65], using a small amount of sorbent (5 mg). However, it was proved that the preparation of MNPs coated with a layer of silica functionalized at the same time with C18 groups and MWCNTs significantly improved the extraction efficiency of zearalenone and its metabolite compounds [65].

Conversely, graphene has also been extensively used as coating material of MNPs. In this sense, graphene MNPs have been successfully applied as MSPE sorbent for the extraction of aflatoxins from cereal samples [66] and patulin from apple juice [67]. Additionally, zeolites have also been used in the preparation of MNPs. For instance, they have been applied for the extraction of four aflatoxins in milk samples [68], obtaining recovery values in the range 79–102% using 90 mg of the designed sorbent. In contrast, Huang et al. also used zeolites to prepare MNPs, but in this case they only needed 1 mg of sorbent to achieve the extraction of domoic acid from seafood samples, achieving high recovery values (93–102%) [69]. On the other hand, Zhang et al. also proposed a MSPE procedure to extract domoic acid from shellfish samples [70]. However, for this purpose, they prepared silica coated MNPs functionalized with a MOF as sorbent. They also used 1 mg of the synthesized sorbent to achieve the extraction of the target analyte, obtaining recovery values in the range 91–107%, which are very similar to the ones reported in the previous work [69].

It also worth highlighting the recent work developed by González-Jartín et al., in which 25 different magnetic nanostructures materials with different coatings (including silica, activated carbon, bentonite, aluminum oxide, GO and biopolymers, among others) were synthesized with differentiated sizes and compositions in order to evaluate them as MSPE sorbents for the extraction of 7 different types of mycotoxins from beer samples [71]. It was observed that MNPs composed of mixtures of alginate and activated carbon or pectin had the highest extraction efficiency.

Magnetic MIPs have also been developed for the selective extraction of aflatoxins and ochratoxins from different food matrices (Table 2). For instance, Wu et al. prepared an MNP with a core of nanoporous carbon magnetized with Co_3_O_4_, which was coated with a dummy MIP for aflatoxins [72]. Ethyl-3-coumarincarboylate was used as template. The best extraction conditions involved using 80 mg of sorbent to achieve the extraction of aflatoxins from corn samples, obtaining recovery values ranging from 75 to 99%. Tan et al. also developed a magnetic dummy MIP for the determination of aflatoxins from corn and tea leaves [73]. In this case, the magnetic core was of Fe_3_O_4_ which was first coated with silica and afterwards the silanol groups of the silica surface were modified with the MIP. This material proved to be superior to the previous one [72], since with only 10 mg of sorbents the recovery values obtained for the four aflatoxins were in the range 76–95% [73], which is very similar to the one described above [72]. Additionally, the chromatographic method was developed by ultra-high performance liquid chromatography (UHPLC), achieving the separation of the analytes in less than 5 min [73], whereas the chromatographic method developed by Wu et al. took longer than 10 min [72].

On the other hand, a magnetic MIP for ochratoxins was developed by functionalizing the MIP on the surface of an MNP coated with polydopamine [74]. Ochratoxin A was used as template. This material was applied for the extraction of ochratoxins from rice and wine samples. Under optimal experimental conditions using 15 mg of the sorbent the recovery values obtained range from 71% to 88% and good sensitivity of the method was achieved (LODs 0.0018–0.018 µg/L). Turan and Sahin also prepared a magnetic MIP for the selective extraction of ochratoxin A from grape juice [75]. In this case, they first prepared bromine-coated MNPs and afterwards these were functionalized with the MIP, which was synthesized by using ochratoxin A as template. High recovery (97%) of ochratoxin A was achieved by just using 5 mg of sorbent in the extraction procedure, showing its great selectivity towards the target analyte.

### 3.6. Micro-Solid-Phase Extraction (µ-SPE)

**Figure 7:**
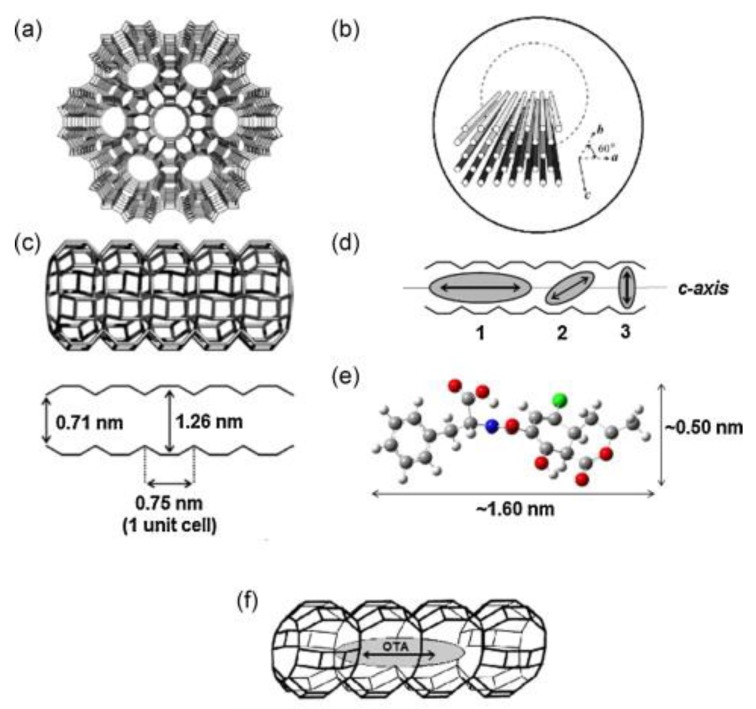
(**a**) Top view of the structure of zeolite Linde Type L (LTL) illustrating its hexagonal framework. It shows a channel surrounded by six neighboring channels. (**b**) Schematic view of some channels in a hexagonal zeolite LTL crystal with cylinder morphology. (**c**) Side view of a channel that consists of 0.75 nm long unit cells with a van der Waals opening of 0.71 nm at the smallest and 1.26 nm at the widest place. (**d**) Schematic illustration of different orientation of molecules in the main channel of LTL. (**e**) The width and length of ochratoxin A. (**f**) Schematic illustration of ochratoxin A in the main channel of LTL (reprinted with permission from reference [76]).

Lee et al. also used µ-SPE for the extraction of ochratoxin A in coffee and cereals, but in this case using zeolite-L crystals enclosed in a porous membrane sheet as sorbent material [76] instead of the commercial MIP described before [41]. Zeolites are crystalline microporous aluminosilicates with an open framework and exchangeable cations, which leads to the presence of pores and cavities (3–10 Å). Their specific size and pore shape allow them to act as sorbents, since they can control the access of molecules with different shape and polarity into their pores (Figure 7). In this work [76], several zeolites (nanosized, road, cylinder and needle) were synthesized with different morphology, length, diameter and particle size, and all of them were evaluated as sorbents in µ-SPE. Among them, the cylinder zeolite was found to be more effective in the extraction of ochratoxin A. Under the optimized conditions, the extraction procedure took 30 min and the elution of the analyte 5 min. The chromatographic method by HPLC-FLD was the same than the one used in their previous work [41]. Moreover, the results were very similar either using zeolites or the commercial MIP as µ-SPE sorbent, since recovery values (92–101%) and LODs (0.09–0.3 µg/Kg) obtained [76] were similar to the ones reported before for the µ-SPE work using MIP [41]. Therefore, zeolite showed good extraction efficiency and selectivity towards ochratoxin A in a similar way to the commercial MIP sorbent, which is more expensive, whereas the preparation of zeolites is simple and they are affordable starting materials. Thus, zeolites can be used as an alternative cost-effective sorbent material.

### 3.7. Solid-Phase Microextraction (SPME)

**Figure 8:**
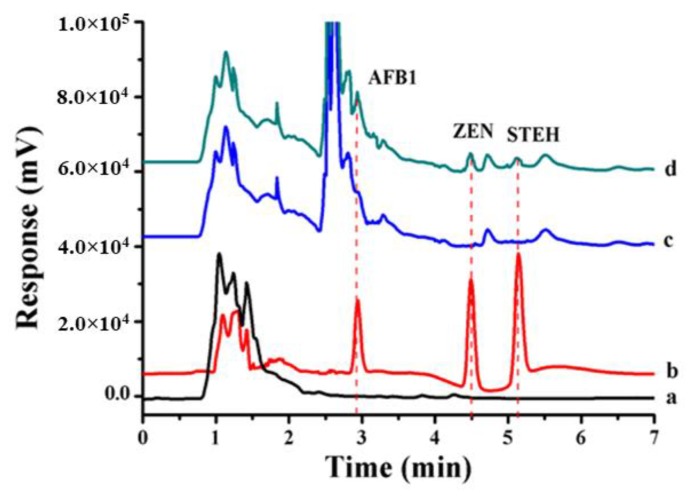
Chromatograms of three mycotoxins obtained from spiked rice grain samples: (**a**) blank sample by in-tube SPME-HPLC method; (**b**) in-tube SPME–HPLC from spiked rice grain sample; (**c**) direct injection mode of HPLC of the blank rice grain sample; (**d**) direct injection mode of HPLC of the spiked rice grain sample. The analytes of three mycotoxins were spiked respective at 0.1 mg/Kg, the volume for direct injection was 20 μL. (Reprinted with permission from reference [79]).

Regarding the analysis of natural toxins, several strategies for preparing SPME fibers have been employed for the extraction of several mycotoxins from different food matrices. For instance, Zhang et al. prepared a carbon-tape SPME fiber for the extraction of ochratoxin A from cheese combined with its analysis by HPLC-MS/MS [77]. The results indicated good extraction efficiency (recovery 93%), and the LOD was 1.5 µg/L, which was higher than other LODs reported for the analysis of ochratoxin A by HPLC-MS/MS [49,50]. On the other hand, Es’haghi et al. proposed a new SPME fiber format by the sol–gel synthesis of functionalized MWCNTs that were introduced inside a polypropylene hollow fiber as the sorbent [78]. In this way, during extraction, the analytes and the sorbent are in contact with each other through the fiber wall pores which act like channels. The prepared hollow fiber was applied for the extraction of two aflatoxins (B1 and B2) from rice and wheat samples. Recoveries achieved in the rice sample were low, ranging from 47% to 54%, whereas the ones obtained for the wheat sample were higher (83–103%). The analysis of the sample extracts was performed by HPLC coupled to a photodiode array detector (HPLC-PDA). Aflatoxins were separated in 10 min and the method exhibited good sensitivity with LODs values in the range 0.061–0.074 µg/L. In addition, more recently, an in-tube SPME strategy has been proposed for the simultaneous multicomponent extraction of three different mycotoxins (aflatoxin B1, zearalenone and sterigmatocystin) from rice samples [79]. For this purpose, a poly(methacrylic acid-co-divinyl-benzene) monolithic column was prepared and applied as SPME fiber for the extraction of the target analytes, which were subsequently analyzed by HPLC-PDA. Extraction was successful, resulting in good recovery values (78–103%) thanks to the effective hydrophobic, π-π and hydrogen bonding interactions established between the monolithic column and the analytes. Additionally, the proposed methodology proved its high potential as clean-up and preconcentration technique (elimination of the matrix interferences) through its comparison with direct HPLC analysis of a rice sample not subjected to the in-tube SPME method developed (Figure 8). However, LODs were higher (0.69–2.03 µg/L) than the ones obtained by MS or in the work previously described for the analysis of aflatoxins by HPLC-PDA [80]. Nonetheless, the separation of the three mycotoxins was achieved in less than 6 min and recovery values were successful for all the analytes assayed [79].

### 3.8. Stir-Bar Sorptive Extraction (SBSE)

**Figure 9:**
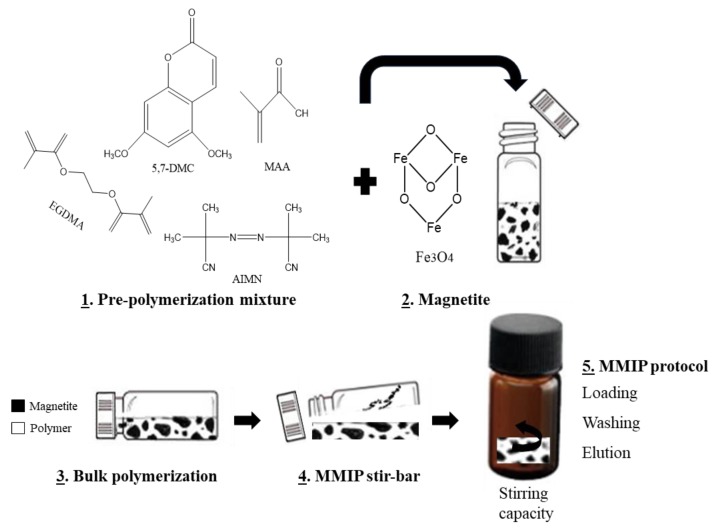
Steps of fabrication and application of the magnetic molecularly imprinted stir-bars (reprinted with permission from reference [81]).

Stir-Bar Sorptive Extraction (SBSE) was proposed by Baltussen et al. [80] in 1999 as a technique to reduce the generation of wastes and to overcome the limited extraction capacity of SPME fibers. This sorptive technique fulfills the GAC requirements, since it is a solvent-less sample preparation method based on a distribution between the sample and a non-miscible liquid phase, similar to the SPME methodology. Nevertheless, instead of using a coated fiber, in SBSE a stir bar is coated, and the amount of non-miscible liquid phase coated is 50–250 times larger than on SPME. For this reason, the extraction ability of SBSE is higher, being able to provide better recovery values than SPME. Conventional SBSE involves using PDMS polymer to coat the glass stir bar, which provides hydrophobic interactions with the target compounds. However, due to the hydrophobic nature of the polymer, it fails to achieve good performance towards the extraction of polar and slightly polar compounds. For this reason, the development of new coating materials has become an important issue to improve SBSE methodologies. In this sense, Diaz-Bao et al. proposed the synthesis of a magnetic dummy MIP stir bar to achieve the SBSE extraction of 5 aflatoxins from milk and cereal-based baby foods combined with LC-MS/MS analysis [81]. Fe_3_O_4_ nanoparticles were first prepared by a co-precipitation method and afterwards they were embedded into a dummy MIP through a bulk polymerization process. Template bleeding is one of the drawbacks of using MIPs, but it can be solved using dummy templates. Thus, in this work, due to the toxicity of aflatoxins, 5,7,-dimethoxycoumarin was used as dummy template. The MIP was successfully grafted to the surface of the MNPs and the polymer was formed around pieces of magnetite, resulting in a monolithic bar with stirring capacity (Figure 9). Once the stir bar was prepared, it was used to perform SBSE of the samples. The extraction procedure was carried out for 45 min and the elution step took another 45 min, which is a considerably long time in comparison with other sample preparation techniques, such as µ-MSPE. In addition, the subsequent chromatographic analysis of the sample extract took 15 min. Moreover, with this technique, low recovery values were obtained, ranging from 39% to 60%. However, very low LODs were achieved, in the range 0.3–1.7 ng/Kg.

## 4. Future Challenges in the Integration of New Advanced Materials on Microextraction Techniques for the Analysis of Natural Toxins in Food Samples

In recent years, the occurrence of other natural toxins which can be found in food products, such as pyrrolizidine, tropane and opioid alkaloids, has aroused great interest [4,7,15,16,17]. The EFSA is currently demanding more information and studies about the presence of these toxic compounds in food in order to know the real exposure of the population to these toxins through their intake because of their potential harmful risks to human health [4,7,8,9,10,11,12]. In this sense, in recent years, several analytical methods have been proposed to identify and quantify these compounds in different food samples [82,83,84]. However, most of these methods employ commercial materials as sorbents and, in addition, none of them have been performed under microextraction conditions. Indeed, only two works have reported the microextraction of opiates by MEPS and µ-MSPE, but from biological samples (blood and human hair, respectively) instead of food [85,86]. Regarding the development of new materials for the extraction of these compounds, apart from the MNPs functionalized with silane groups which were prepared as sorbent for the microextraction of morphine from human hair [86], different MIP materials have been synthesized for the selective extraction of tropane alkaloids [87,88,89,90]. In these works, MIPs for the extraction of atropine and scopolamine have been applied as sorbents in pharmaceutical formulations [87,88] and biological samples (urine and plasma samples) [89], except the work published by Zeng et al., which employs a MIP for the extraction of four tropane alkaloids (including scopolamine, atropine, anisodine and anisodamine) from fruit samples [90], which was the only one applied for food sample preparation. This MIP was prepared by precipitation polymerization using anisodine as a template. Once it was prepared, 100 mg of the material were packed in a SPE cartridge and the target analytes were extracted according to a traditional SPE procedure. Afterwards, the resulting purified sample extracts were analyzed by HPLC-MS/MS, achieving good recovery values (82–102%) and LODs in the range 2.12 to 0.52 µg/L. However, due to the amount of sorbent used it cannot be considered a miniaturized SPE extraction. In contrast, no articles were found in the literature for the microextraction of pyrrolizidine alkaloids from food products, nor about the development of new materials for the extraction of these compounds. Therefore, future trends in food sample preparation should focus on the development of potential analytical procedures, which involve both the development of advanced materials and their integration as sorbent in microextraction techniques for their application to the extraction of these natural alkaloids from food samples. This is practically an unexplored path that is of great interest due to the harmful effect that the intake of these compounds can impose for human health, which may also help to expand knowledge about them.

## 5. Conclusions

As shown throughout this review, the integration of new materials as sorbents in microextraction techniques is a powerful approach to develop food sample preparation procedures which enables to meet the GAC requirements. By scaling down the procedures, it is possible to develop less time-consuming and more cost-effective analytical methods to extract natural toxins from food samples. In this sense, SPME was one of the first microextraction techniques which were developed. However, in recent years, its use has decreased, and new emerging techniques have appeared, like MEPS, µ-SPE, PT-SPE and in-syringe SPE. In contrast, other traditional sample preparation procedures have evolved into more sophisticated and environmentally friendly modes through their miniaturization (such as m-SPE and µ-dSPE). Among the microextraction techniques employed for the determination of natural toxins in food samples, SPME and SBSE have been the ones with worst extraction efficiency, providing low recovery values. Nonetheless, the effectiveness of SPME improves when it is used in the in-tube mode. On the other hand, µ-MSPE has been the extraction technique most widely used due to its simplicity and high extraction potential, followed by m-SPE. In this context, dispersive-based microextraction techniques have proved to be very suitable to perform multicomponent extractions, since the interaction is higher between the analytes and the sorbent than when the sorbent is packed inside a cartridge.

Regarding the development of new advanced materials as novel sorbents for the extraction of natural toxins from food samples, they have offered new opportunities for improving analytical methods, as it has been shown through the comparison of some of them with commercially available sorbent materials. Moreover, the use of graphene as sorbent in different microextraction techniques has aroused great interest due to its advanced properties, like huge surface area, good chemical and mechanical stability, good adsorption capability, and possibility of surface modification. In this sense, it has been used as a sorbent in several microextraction techniques, including m-SPE, in-syringe SPE, PT-SPE, µ-dSPE and µ-MSPE for the extraction of different analytes. Magnetic materials have also proved their superiority as potential sorbents, enabling to develop simple, effective and quick extraction methods. On the other hand, the development of MIPs has proved to be very suitable to achieve the selective extraction of different compounds, providing effective clean-up of matrix interferences. Additionally, it is worth highlighting that it is more convenient to prepare dummy MIPs to avoid false positive results, and thus develop more reliable methods. However, MIPs are not suitable for performing multicomponent extractions due to their high selectivity. Therefore, for multicomponent extractions is better to use a sorbent material with high versatility able to provide different types of interaction, like for instance mixed-mode or bi-functionalized sorbent materials.

Overall, great advances have been made in the food sample preparation field in the last decade, involving the development of new materials and their integration in microextraction techniques. Among all the natural toxins reviewed, mycotoxins have been by far the most studied in recent years. Nevertheless, the development of advanced analytical strategies for the determination of other natural toxins such as pyrrolizidine, tropane and opioid alkaloids through the combination of new materials and microextraction techniques is still a great challenge. In this sense, the future trends for the next years in this field should focus on the development of quick multicomponent methods to achieve the determination of these unexplored compounds in food samples by integrating advanced materials as sorbents in microextraction procedures. In addition, there is still a long way to really achieve analytical methods meeting all the GAC requirements. As showed in the articles reviewed, despite decreasing the amount of sorbent, many of them still using large amounts of sample and solvents in the sample pretreatment. Additionally, reducing the elution volume is also necessary. Although it is true that there have been more efforts to reduce the solvent volume for the elution step than for the extraction. Therefore, in order to truly achieve miniaturized and microextractive methods, efforts must be made in the future not only to improve the sorbent-based microextraction part, but also the previous part. This way, sensitive and environmentally friendly analytical strategies would be achieved, contributing to improve food safety by using low consumption of samples, solvents and time.

## Figures and Tables

**Table 1:** Application of sorbent-based microextraction techniques for isolation of natural toxins in food samples (2009–2019).

Food Matrix (Amount)	Analytes	Sample Pretreatment	Microextraction Technique	Analysis	Recovery (%)	LOD	Ref.
Cereal flours (2 g)	AF (B1, B2, G1, G2)	Extraction with 10 mL of MeOH/phosphate buffer (80/20, *v*/*v*, pH 5.8). Evaporation to dryness and reconstitution with 4 mL of phosphate buffer. An aliquot of the extract (2 mL) subject to microextraction.	SPME Sorbent: Commercial fibers Elution: 0.1 mL MeOH	HPLC-FLD	49–59	0.035-0.2 μg/Kg	[34]
Nuts, cereals, dried fruits and spices (0.5 g)	AF (B1, B2, G1, G2)	Extraction with 1 mL of MeOH/H_2_O (80/20, *v*/*v*). An aliquot of the extract (0.1 mL) mixed with 0.1 mL of 50 mM Tris buffer and brought to a total volume of 1 mL with H_2_O before microextraction.	In-tube SPME * Sorbent: SUPEL-Q PLOT capillary	HPLC-MS	81–109	0.0021-0.0028 μg /L	[35]
Fruit juice and dried fruit (1 mL)	PAT	-	In-tube SPME * Sorbent: Carboxen-1006 PLOT capillary	HPLC-MS	> 92	0.023 μg /L	[36]
Nut and grain samples (0.5 g)	OTA, OTB	Extraction with 1 mL of MeOH/H_2_O (80/20, *v*/*v*). Defatted with 3 mL hexane, supernatant discarded. An aliquot of the clean extract (0.1 mL) brought to a total volume of 1 mL with H_2_O before microextraction.	In-tube SPME * Sorbent: Carboxen-1006 PLOT capillary	HPLC-MS	88	0.089-0.092 μg /L	[37]
Wine (0.05 mL)	OTA	-	In-tube SPME * Sorbent: Luna C18 particles	HPLC-MS/MS	61–73	0.02 μg/L	[38]
Powdered infant milk (3 mL) and mineral waters (50 mL)	ZEN, α-ZAL, β-ZAL, α-ZEL, β-ZEL, ZAN	Extraction of milk samples with 0.15 mL acetic acid and 6 mL ACN. Evaporation up to 2.5 mL and reconstitution with H_2_O to 25 mL, pH adjusted to 3.0 before microextraction.	µ-dSPE Sorbent: 80 mg of MWCNTs Elution: 30 mL MeOH/Acetone (1/1, *v*/*v*)	HPLC-MS/MS	77–120	0.05–2.02 µg/L	[39]
Peach seed, milk powder, corn flour (0.2 g) and beer (0.2 mL)	AF (B1), OTB, T-2, OTA, ZEN	Microwave assisted extraction of solid samples with 0.2 g NaCl and 5 mL MeOH/H_2_O (70/30, *v*/*v*). An aliquot of the extract (0.2 mL) brought to a total volume of 5 mL with H_2_O before microextraction. Liquid samples diluted with H_2_O up to 5 mL before microextraction.	µ-dSPE Sorbent: 12.5 µg zirconia nanoparticles Elution: 0.1 mL MeOH	UHPLC-MS/MS	84–105	0.0022–0.017 µg/L 0.0036–0.033 μg/Kg	[40]
Coffee (10 g) and grape juice (10 mL)	OTA	Extraction of coffee samples with 100 mL of carbonate. An aliquot of the extract (10 mL) adjusted to pH 1.5 before microextraction. Grape juice samples adjusted to pH 1.5 before microextraction.	µ-SPE Sorbent: 15 mg AFFINIMIP^TM^ OTA Elution: 0.25 mL MeOH/Acetic acid (98:2, *v*/*v*)	HPLC-FLD	91–101	0.02–0.06 μg/Kg	[41]
Wine (0.35 mL)	OTA	-	MEPS Sorbent: 4 mg C18 sorbent Elution: 0.05 mL ACN/2% Acetic Acid (90/10, *v*/*v*)	HPLC-FLD	76–108	0.08 μg/L	[42]

* Elution performed with mobile phase (online system); ACN: Acetonitrile; AF: Aflatoxin; F: Fumonisin; HPLC-FLD: High performance liquid chromatography coupled to fluorescence; HPLC-MS/MS: High performance liquid chromatography coupled to tandem mass spectrometry; HPLC-MS: High performance liquid chromatography coupled to mass spectrometry; MeOH: Methanol; MEPS: Microextraction by packed sorbent; MWCNTs: Multiwalled carbon nanotubes; OTA: Ochratoxin A; OTB: Ochratoxins B; PAT: Patulin; SPME: Solid-phase microextraction; T-2: T-2 toxin; UHPLC-FLD: Ultra High performance liquid chromatography coupled to fluorescence; UHPLC-MS: Ultra High performance liquid chromatography coupled to tandem mass spectrometry; ZAL: Zearalanol; ZAN: Zearalanone; ZEL: Zearalenol; ZEN: Zearalenone; µ-dSPE: Micro-dispersive solid-phase extraction; µ-SPE: Micro-solid-phase extraction.

**Table 2:** Application of new advanced materials on sorbent-based microextraction techniques to isolate natural toxins from food samples (2009–2019).

Food Matrix (Amount)	Analytes	Sample Pretreatment	Microextraction Technique	Analysis	Recovery (%)	LOD	Ref.
Cereals (5 g)	AF (B1, B2, G1, G2)	Extraction with 25 mL of MeOH/H_2_O (80/20, *v*/*v*). Evaporation of the methanolic fraction of an aliquot of the extract (15 mL). Addition of Britton-Robinson buffer (pH 5.2) up to 3 mL. An aliquot of the extract (2 mL) subject to microextraction.	m-SPE Sorbent: 50 mg hyperbranched polymer Elution: 0.2 mL ACN	HPLC-FLD	83–103	0.012–0.120 μg/Kg	[48]
Apple juice (1 mL)	PAT	-	m-SPE Sorbent: 30 mg CD-based polymers Elution: 1 mL Diethyl ether/ACN (4/1, *v*/*v*)	HPLC-DAD	n.p.	n.p.	[49]
Apple juice (1 mL)	PAT	Dilution with 1 mL of H_2_O before microextraction.	m-SPE Sorbent: 50 mg SiO_2_maleicpolymer@MIP Elution: 5 mL de acidified ACN	HPLC-DAD	82–98	8.6 µg/L	[50]
Apple, apple juice, hawthorn, hawthorn juice, mixed juice, wines and tomato (10 g)	PAT	Extraction with 10 mL of ACN, 4 mg MgSO_4_ and 1 g NaCl. An aliquot of the extract (1 mL) evaporated to dryness and reconstituted with 1 mL H_2_O before microextraction.	m-SPE Sorbent: 30 mg dual dummy-MIP Elution: 3 mL MeOH	HPLC-MS/MS	81–106	0.05–0.2 μg/Kg	[51]
Bell pepper, rice and corn flakes (1 g)	F (B1, B2, B3)	Extraction with 6 mL ACN/H_2_O (84/16, *v*/*v*). An aliquot of the extract (1 mL) evaporated to dryness and reconstituted with 1 mL ACN/H_2_O (90/10, *v*/*v*) before microextraction.	m-SPE Sorbent: 20 mg MIP Elution: 1 mL MeOH/Acetic acid (95/5, *v*/*v*)	HPLC-MS/MS	62–86	4.5–44 µg/Kg	[52]
Maize, barley and oat (5 g)	T-2	Extraction with 25 mL of ACN/H_2_O (84/16, *v*/*v*). For oat samples, after the solid-liquid extraction, the extract was additionally defatted with 10 mL of hexane. An aliquot of the sample extracts (1 mL) evaporated to dryness and reconstituted with 1 mL MeOH/H_2_O (20/80, *v*/*v*) before microextraction.	m-SPE Sorbent: 50 mg MIP Elution: 3 mL MeOH/Acetic acid (95/5, *v*/*v*)	HPLC-MS/MS	60–73	0.4–0.6 µg/Kg	[53]
Milk (1 mL)	AF (B1, M1), OTA, ZEN, α-ZEL, β-ZEL, ZAN, α-ZAL, β-ZAL	Extraction with 5 mL ACN with 0.1% formic acid. Supernatant of the extract evaporated to dryness and reconstituted with 0.5 mL ACN/H_2_O (20/80, *v*/*v*) and diluted up to 5 mL with 5 mL of H_2_O before microextraction.	m-SPE Sorbent: 10 mg rGO/Au Elution: 5 mL MeOH/ACN/Formic acid (50/49/1, *v*/*v*/*v*)	UHPLC-MS/MS	70–111	0.01–0.07 ng/mL	[54]
Soy-based foods (2 g)	AF (B1, B2, G1, G2)	Extraction with 10 mL ACN/H_2_O (75/25, *v*/*v*). Diluted up to 50 mL with 10% NaCl aqueous solution before microextraction.	In syringe SPE Sorbent: 30 mg 3DG@Fe_3_O_4_ Elution: 0.7 mL MeOH	HPLC-FLD	83–103	0.09–0.15 µg/Kg	[55]
Soy-based foods (2 g)	AF (B1, B2, G1, G2)	Extraction with 10 mL ACN/H_2_O (75/25, *v*/*v*). Diluted up to 50 mL with 7% NaCl aqueous solution before microextraction.	In syringe SPE Sorbent: PU/GO nanofibers Elution: 0.75 mL MeOH	HPLC-FLD	76–101	0.09–0.15 µg/Kg	[56]
Maize (5 g)	AF (B1, B2, G1, G2)	Extraction with 20 mL ACN/H_2_O (80/20, *v*/*v*). Evaporation to dryness and reconstituted with 0.1 mL MeOH. Diluted up to 10 mL with H_2_O before microextraction.	In syringe SPE Sorbent: 15 mg β-CDPG Elution: 2 mL MeOH/DCM (2/1, *v*/*v*)	HPLC-FLD	91–105	0.0075–0.030 μg/Kg	[57]
Shellfish (0.2 g)	YTX, OA, DTX (1), GYM, SPX (1), PTX (2), AZA (1)	Extraction with 9 mL MeOH. An aliquot of the extract (0.1 mL) evaporated to dryness and reconstituted with 0.2 mL H_2_O before microextraction.	PT-SPE Sorbent: 2 mg graphene Elution: 2 mL ACN with 0.5% ammonium hydroxide (for basic conditions) or with 0.5% formic acid (for acid conditions)	HPLC-MS/MS	78–90	0.1–1.5 μg/Kg	[58]
Peanut (50 g)	AF (B1, B2, G1, G2)	Extraction with MeOH/H_2_O (80/20, *v*/*v*). An aliquot of the extract (8 mL) diluted with H_2_O before microextraction.	µ-dSPE Sorbent: 5 mg GO Elution: 2 mL MeOH	HPLC-FLD	85–101	0.08–0.65 μg/Kg	[59]
Milk and yogurt (1.5 mL)	ZEN, α-ZEL, β-ZEL, ZAN, α-ZAL, β-ZAL	Extraction of milk samples with 3 mL ACN and 0.075 mL acetic acid. Evaporation of the supernatant until 1.5 mL and diluted with H_2_O up to 25 mL, pH adjusted to 7 before microextraction. Extraction of yogurt samples with 4.5 mL and 0.075 mL acetic acid. The rest of the procedure the same as for milk samples.	µ-MSPE Sorbent: 80 mg Fe_3_O_4_@pDA Elution: 8 mL MeOH	HPLC-MS/MS	70–120	0.21–4.77 µg/L	[60]
Mineral and tap water (25 mL)	ZEN, α-ZEL, β-ZEL, ZAN, α-ZAL, β-ZAL	Adjustment of pH to 7 before microextraction.	µ-MSPE Sorbent: 60 mg Fe_3_O_4_@pDA NPs Elution: 6 mL MeOH	HPLC-MS/MS	70–119	0.02–1.1 µg/L	[61]
Red wine (50 mL)	AF (B1, B2, G1, G2)	-	µ-MSPE Sorbent: 4.4 mg PD-MNPs Elution: 0.25 ACN/MeOH (1/1, *v*/*v*)	HPLC-MS/MS	97–108	0.0012–0.0031 µg/L	[62]
Milk and dairy products (5 mL)	AF (M1)	Extraction with 5 mL hexane and 5 mL MeOH/2 mM NaCl aqueous solution (8/2, *v*/*v*) before microextraction.	µ-MSPE Sorbent: 8 mg AMNPs Elution: 2 mL DCM/MeOH/Acetic acid (80/19/1, *v*/*v*/*v*)	HPLC-FLD	97–116	0.2 ng/L	[63]
Shellfish (2 g)	AZA (1, 2, 3), OA, DTX (1, 2)	Extraction with 10 mL MeOH/H_2_O (4/1, *v*/*v*). The supernatant mixed with 2 mL hexane, evaporated until 1 mL and addition of 4 mL of H_2_O before microextraction.	µ-MSPE Sorbent: 50 mg MMM Elution: 2 mL Formic acid/MeOH (5/95, *v*/*v*)	UHPLC-MS/MS	83–119	0.4–1.0 μg/Kg	[64]
Maize (6 g)	ZEN, α-ZEL, β-ZEL, ZAN, α-ZAL, β-ZAL	Extraction with 24 mL of ACN/H_2_O (75/25, *v*/*v*). The extract diluted up to 25 mL with H_2_O before microextraction.	µ-MSPE Sorbent: 5 mg MNPs-MWCNT-nanoC18 Elution: 1 mL ACN	HPLC-MS	92–98	0.6–1.0 μg/mL	[65]
Rice, wheat and sesame (50 g)	AF (B1, B2, G1, G2)	Extraction of rice and wheat samples with 200 mL Acetone/H_2_O (50/50, *v*/*v*). Elimination of the acetone fraction before microextraction. Extraction of sesame samples with 100 mL hexane and 200 mL Acetone/H_2_O (50/50, *v*/*v*). The rest of the procedure the same as for rice and wheat samples.	µ-MSPE Sorbent: 10 mg MGNP Elution: 2 mL Acetone/H_2_O (1/1, *v*/*v*)	HPLC-FLD	64–122	0.025–0.075 µg/Kg	[66]
Apple juice (5 g)	PAT	Extraction with 5 mL ethyl acetate/hexane (96/4, *v*/*v*), 1 g NaH_2_PO_4_ and 5 g Na_2_SO_4_. An aliquot of the organic phase (3 mL) mixed with 0.02 mL acetic acid, evaporated to dryness and reconstituted with 2 mL H_2_O at pH 6.2 before microextraction.	µ-MSPE Sorbent: 30 mg MGO Elution: 1 mL ACN	HPLC-UV	69–83	2.3 μg/Kg	[67]
Milk (20 mL)	AF (B1, B2, G1, G2)	-	µ-MSPE Sorbent: 90 mg M/ZIF-8 Elution: 1 mL ACN/DCM (1/1, *v*/*v*)	UHPLC-MS/MS	79–102	2.3–8.1 ng/L	[68]
Seafood (5 g)	DA	Extraction with 20 mL MeOH/H_2_O (1/1, *v*/*v*). The resultant sample extract subjected to microextraction.	µ-MSPE Sorbent: 1 mg Fe_3_O_4_ SPs@ZIF8/Zn^2+^ Elution: 0.4 mL 3 mM histidine solution	HPLC-MS/MS	93−102	0.2 ng/L	[69]
Shellfish samples (5 g)	DA	Extraction with 20 mL MeOH/H_2_O (1/1, *v*/*v*). The resultant sample extract brought to a total volume of 25 mL with MeOH/H_2_O (1/1, *v*/*v*) before microextraction.	µ-MSPE Sorbent: 1 mg Fe_3_O_4_@SiO_2_@UiO-6 Elution: 1.5 mL ACN with 20% acetic acid	HPLC-MS/MS	91–107	1.45 µg/L	[70]
Beer (6 mL)	DON, ZEN, AF (B1, B2, G1, G2), F (B1)	Clean-up with a C18 sorbent. An aliquot of the clean sample (0.1 mL) evaporated to dryness and reconstituted with 0.48 mL ACN/H_2_O/acetic acid (49/50/1, *v*/*v*/*v*) before microextraction.	µ-MSPE Sorbent: 25 mg MNM Elution: 0.5 mL ACN/H_2_O/acetic acid (79/20/1, *v*/*v*/*v*)	UHPLC-MS/MS	87	n.p.	[71]
Corn (25 g)	AF (B1, B2, G1)	Extraction with 5 g NaCl and 125 mL MeOH/H_2_O (7/3, *v*/*v*). An aliquot of the extract (15 mL) mixed with 45 mL of PBS before microextraction.	µ-MSPE Sorbent: 80 mg MNPC Elution: 1.2 mL ACN/H_2_O (6/4, *v*/*v*).	HPLC-FLD HPLC-MS/MS	75–99	0.05–0.07 µg/L	[72]
Tea leaves and corn (5 g)	AF (B1, B2, G1, G2)	Extraction with 10 mL ACN/H_2_O (60/40, *v*/*v*). 5 mL of the extract subjected to microextraction.	µ-MSPE Sorbent: 10 mg MMIP Elution: 1 mL ACN/formic acid (95/5, *v*/*v*).	UHPLC-MS/MS	76–95	0.05–0.1 μg/Kg	[73]
Rice (25 g) and wine (20 mL)	OTA, OTB, OTC	Extraction of rice samples with 100 mL ACN/H_2_O (60/40, *v*/*v*) before microextraction. Wine samples diluted up to 25 mL with a solution of 2.5 M NaCl and 0.24 M NaHCO_3_ before microextraction.	µ-MSPE Sorbent: 15 mg Fe_3_O_4_@PDA MIPs Elution: 1 mL ACN	HPLC-FLD	71–88	0.0018–0.018 µg/Kg	[74]
Grape juice	OTA	-	µ-MSPE Sorbent: 5 mg MMIP Elution: -	UV–vis	97	0.374 mg/L	[75]
Coffee (10 g) and cereals (5 g)	OTA	Extraction with 10 mL 1% carbonate aqueous solution. Sample extract adjusted to pH 1.5 before microextraction.	µ-SPE Sorbent: 10 mg LTL Elution: 0.4 mL MeOH	HPLC-FLD	92–101	0.09–0.3 μg/Kg	[76]
Cheese (0.05 g)	OTA	-	SPME Sorbent: Carbon-tape fiber Elution: 0.15 mL MeOH	HPLC-MS/MS	93	1.5 μg/L	[77]
Rice and wheat (10 g)	AF (B1, B2)	Extraction with 1 g NaCl and 100 mL MeOH/H_2_O (80/20, *v*/*v*). Evaporation of the methanolic fraction of the extract and diluted with 40 mL H_2_O. An aliquot of the extract (25 mL) subject to microextraction.	SPME Sorbent: 50 mg CNT Elution: 2 mL MeOH	HPLC-DAD	47–103	0.061–0.074 μg/L	[78]
Rice (2 g)	AF (B1), ZAN, STEH	Extraction with 10 mL ACN/MeOH/H_2_O (51/9/40, *v*/*v*/v), 1.5 g MgSO_4_ and 0.5 g NaCl. Evaporation to dryness and reconstituted with 3 mL 0.1% TFA/ACN (99/1, *v*/*v*) before microextraction.	SPME in-tube * Sorbent: MAA-co-DVB Elution:-	HPLC-PDA	78–103	0.69–2.03 μg/Kg	[79]
Milk (1 g) and baby foods (3 g)	AF (B1, B2, G1, G2, M1)	Extraction of milk samples with 3 mL 1% formic acid solution. Supernatant discarded and solid residue extracted with 6 mL chloroform. Evaporation to dryness and reconstitution with 4 mL H_2_O before microextraction. Baby food samples dissolved with 1% formic acid solution. Supernatant discarded and solid residue extracted with 18 mL chloroform. Evaporation to dryness and reconstitution with 6 mL H_2_O before microextraction.	SBSE Sorbent: 0.5 g MMIP-SB Elution: 3 mL MeOH/acetic acid (75/25, *v*/*v*)	HPLC-MS/MS	39–60	0.3–1.0 ng/Kg	[80]

* Elution performed with mobile phase (online system); ACN: Acetonitrile; AF: Aflatoxin; AMNPs: Aptamer-functionalized magnetic nanoparticles; AZA: Azaspiracid; CD: Cyclodextrin; CNT: Carbon nanotube; DA: Domoic acid; DAD: Diode array detector; DCM: Dichloromethane; DON: Deoxynivalenol; DTX: Dinophysistoxin; F: Fumonisin; Fe_3_O_4_ SPs@ZIF8/Zn^2+^: Modified magnetic zeolite imidazolate framework-8; Fe_3_O_4_@PDA MIPs: Magnetic polydopamine-based molecularly imprinted polymer; Fe_3_O_4_@pDA NPs: Core–shell polydopamine magnetic nanoparticles; Fe_3_O_4_@SiO_2_@UiO-6: Magnetite@silica core-shell magnetic microspheres; FLD: Fluorescence; GO: Graphene oxide; GYM: Gymnodimine; HPLC: High performance liquid chromatography; LTL: Zeolites linde type; M/ZIF-8: Magnetic zeolite imidazolate framework-8; MAA-co-DVB: Methacrylic acid-co-divinyl-benzene; MeOH: Methanol; MEPS: Microextraction by packed sorbent; MGNP: Magnetic-graphene nanoparticles; MGO: Magnetic graphene oxide; MIP: Molecular imprinted polymer; MMIP: Magnetic molecularly imprinted polymer; MMIP-SB: Magnetic molecularly imprinted stir-bars; MMM: Magnetic mesoporous microspheres; MNM: Magnetic nanostructured materials; MNPC: Magnetic nanoporous carbon; MNPs: Magnetic nanoparticles; MS: Mass spectrometry; MS/MS: Tandem mass spectrometry; m-SPE: Miniaturized solid phase extraction; MWCNTs: Multiwalled carbon nanotubes; n.p.: Not provide; OA: Okadaic acid; OTA: Ochratoxin A; OTB: Ochratoxin B; OTC: Ochratoxin C; PAT: Patulin; PBS: Phosphate buffer saline; PDA: Photodiode array; PD-MNPs: Polydopamine magnetic nanoparticles; PT-SPE: Pipette-tip solid phase extraction; PTX2: Pectenotoxin-2; PU: Polyurethane; rGO: Reduced Graphene oxide; SBSE: Stir-bar sorptive extraction; SPE: Solid-phase extraction; SPME: Solid-phase microextraction; SPX1: Spirolides-1; STEH: Sterigmatocystin; TFA: Trifluoroacetic acid; T-2: T-2 toxin; UHPLC: Ultra high performance liquid chromatography; UV/vis: Ultraviolet/visible; YTX: Yessotoxins; ZAL: Zearalanol; ZAN: Zearalanone; ZEL: Zearalenol; ZEN: Zearalenone; β-CDPG: β-cyclodextrin supported on porous graphene nanohybrid; µ-dSPE: Micro-dispersive solid-phase extraction; µ-MSPE: Micro-magnetic solid-phase extraction; µ-SPE: Micro-solid-phase extraction; 3DG@Fe_3_O_4_: Magnetic three-dimensional graphene sorbent.
